# Book Notices

**Published:** 1867-02

**Authors:** 


### EDITORIAL.

### BOOK NOTICES.

*A Manual of Medical Jurisprudence.* By ALFRED SWAINE TAYLOR, M.D., F.R.S. Sixth American from the eighth and revised London edition, with Notes and References to American Decisions. By CLEMENT B. PENROSE, of the Philadelphia Bar. Philadelphia: Henry C. Lea. 1866.

The merits of this excellent work are too well known to the profession to require any new encomium at this late date. The study of medical jurisprudence is one which should not be neglected, since every practitioner is, at any hour, liable to be called upon for medical testimony of some sort, without warning or opportunity for extended consultation of the authorities. Careful perusal of a work like this will remove from the mind of the physician much of that indefinite dread which so often makes such a mountain of a mole-hill subpœna. "In preparing for the press the present edition it has been thought advisable to restore some portions of the matter omitted by the author, and also to introduce some material from his Principles and Practice of Medical Jurisprudence, thus rendering the volume more complete for those to whom his larger work is not accessible. These additions will be found in the articles on noxious animal food, trichiniasis, sexual malformation, insanity as affecting civil responsibility, suicidal mania and suicide, and life insurance." The illustrations, representing the microscopical appearances of substances used as poisonous agents, add not a little to the value of the chapters which treat of death by poisoning.

For sale by W. B. Keen & Co., 148 Lake Street.

